# Systematic reconstruction of TRANSPATH data into Cell System Markup Language

**DOI:** 10.1186/1752-0509-2-53

**Published:** 2008-06-23

**Authors:** Masao Nagasaki, Ayumu Saito, Chen Li, Euna Jeong, Satoru Miyano

**Affiliations:** 1 Human Genome Center, Institute of Medical Science, University of Tokyo, 4-6-1 Shirokanedai, Minato-ku, Tokyo 108-8639, Japan

## Abstract

**Background:**

Many biological repositories store information based on experimental study of the biological processes within a cell, such as protein-protein interactions, metabolic pathways, signal transduction pathways, or regulations of transcription factors and miRNA. Unfortunately, it is difficult to directly use such information when generating simulation-based models. Thus, modeling rules for encoding biological knowledge into system-dynamics-oriented standardized formats would be very useful for fully understanding cellular dynamics at the system level.

**Results:**

We selected the TRANSPATH database, a manually curated high-quality pathway database, which provides a plentiful source of cellular events in humans, mice, and rats, collected from over 31,500 publications. In this work, we have developed 16 modeling rules based on hybrid functional Petri net with extension (HFPNe), which is suitable for graphical representing and simulating biological processes. In the modeling rules, each Petri net element is incorporated with Cell System Ontology to enable semantic interoperability of models. As a formal ontology for biological pathway modeling with dynamics, CSO also defines biological terminology and corresponding icons. By combining HFPNe with the CSO features, it is possible to make TRANSPATH data to simulation-based and semantically valid models. The results are encoded into a biological pathway format, Cell System Markup Language (CSML), which eases the exchange and integration of biological data and models.

**Conclusion:**

By using the 16 modeling rules, 97% of the reactions in TRANSPATH are converted into simulation-based models represented in CSML. This reconstruction demonstrates that it is possible to use our rules to generate quantitative models from static pathway descriptions.

## Background

Biological pathways are reaction-networks of the biological processes within a cell, which can be classified into three categories: gene regulatory networks, metabolic pathways and signaling pathways. Due to the nature of concurrency of biological pathways, Petri nets have been used to model and analyze biological pathways in many studies [1-9].

Nagasaki *et al*. [10] have proposed an object-oriented Petri nets called hybrid functional Petri net with extension (HFPNe) for modeling complex biological systems. HFPNe-based modeling allows users to easily understand biological networks with a graphical representation and reveals the expressiveness when representing the dynamic behaviors of sophisticated biological pathways. To improve the maintainability and reusability of HFPNe models, we have developed an XML format called the Cell System Markup Language (CSML) [11], with the aim of creating an exchange format for modeling, visualizing, and simulating biological pathways.

Information about biological networks has been accumulated over many years, mainly through experimental analyses. However, there is a growing need for a framework to interpret biological data in an integrated manner and, further, to simulate and predict biological functions beyond simply storing and querying data. It is essential for such a framework to have incorporated the capability to visually represent system dynamics. There are several works to convert biological pathways into Petri net representation [12-15]. However the results are not saved into an exchange format. Although there are several formats to represent biological pathways, such as SBML [16], CellML [17], and BioPAX [18], these formats do not fully support both visualization and simulation of biological pathways. Instead, various tools have been developed to perform these functions independently. However, visualization tools generate images, mostly displayed as static pictures, and save them in noneditable binary files. Simulation tools usually encode model parameters into the programming language. Hence, it is not easy to change those parameters or to integrate them with other resources.

In this paper, we present a method for increasing the overall utilization of the valuable pathway data available in conventional pathway databases.

### Hybrid functional Petri net with extension

CSML is based on HFPNe, a graphical language, for modeling and simulation of biological pathways. Thus, we first describe the basic elements of HFPNe and then how these elements are modeled in CSML. The formal definition of HFPNe is provided in Nagasaki *et al*. [10].

**Figure 1:**
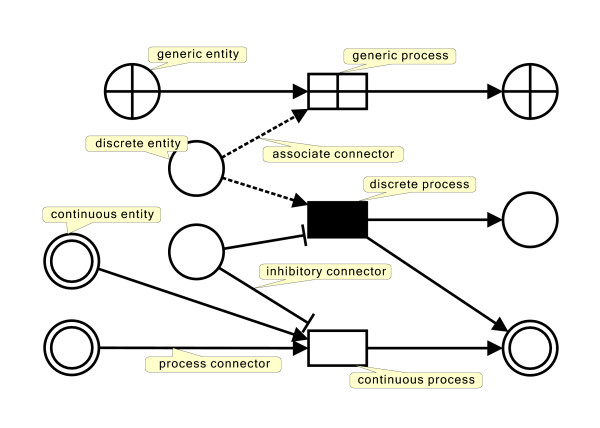
**Graphical notations of HFPNe components**. Redrawn from Nagasaki *et al*. [10] (Figure 1).

• HFPNe consists of two types of nodes, called entities and processes, and three types of connectors – the formal terms in Petri nets are places, transitions, and arcs, respectively. Renaming the elements would make them more intuitive and easier for biologists to understand.

• Entities, represented by circles as a visual component, model biological objects (e.g., mRNAs, proteins, and chemical compounds), conditions (e.g., pH and temperature), states (e.g., the on/off state of a genetic switch), and cellular organelles (e.g., nucleus and cytoplasm). In the case of chemical reactions, the compounds involved usually have specific quantities. In HFPNe, an entity can take any object that can be represented in programming languages like an instance of a class in C++ or Java. In a simple model, an object may take a primitive value such as a positive integer for the copy number of an mRNA and a real value for the concentration of a protein.

• Processes, represented by boxes as a visual component, model events among entities, such as phosphorylation, translocation, and apoptosis. In HFPNe, each process can define any event/function that can be performed by programming languages. In a simple model, the event/function can be the speed of a reaction or a discrete reaction.

• Connectors always connect an entity with a process via a directed edge and describe the relationship between nodes. HFPNe defines three different types of connectors – a process connector, an association connector, and an inhibitory connector – represented by a solid line, a dashed line, and a solid line with a rectangle head as a visual component, respectively. The process connector can be used both as an input and output connector. In addition, it is possible to assign a function to each process connector. Therefore, the values of entities increase or decrease depending on the direction of the process connector and the function defined in the connected process. On the other hand, the values of entities connected with association or inhibitory connectors are not changed. The association connector can be used only as an input connector and is used to enhance the reaction of the process, whereas the behavior of the inhibitory connector is the opposite. All connectors can take values or scripts as a threshold attribute and specify the active state of the process. The graphical notations of HFPNe components are shown in Figure 1.

### Model structure in CSML

The CSML format primarily consists of model, view, biological property, and simulation property. The structure of CSML is as follows: The top-level element in CSML is csml:project, which has an id and a version attribute, and represents a pathway model. The csml:project element contains a single csml:model element. The csml:model element also has an id and a name attribute, and takes the following elements: csml:entitySet, csml:processSet, and csml:factSet; these are sets of csml:entity, csml:process, and csml:fact, respectively. The outline of the CSML structure is shown in Table 1.

**Table 1:** The main structure of CSML.

<csml:project>
<csml:model modelID="" modelVersionID="">
<csml:entitySet>
<csml:entity>*
</csml:entitySet>{0,1}
<csml:processSet>
<csml:process id="" name="" type="">*
<connectorList><connector/>+</connectorList>{0,1}
</csml:processSet>{0,1}
<csml:factSet>
<csml:fact>*
</csml:factSet>{0,1}
<csml:simulationProperty>{0,1}
<csml:biologicalProperty/>{0,1}
</csml:model>
<csml:subModelSet/>
<csml:globalSimulatinProperty/>
<csml:globalViewProperty/>
<csml:globalAnimationProperty/>
<csml:chartSet/>
<csml:viewSet/>
<csml:globalBiologicalProperty/>
<csml:references/>
</csml:project>

The tags explained in the context are shown in black. On the other hand, the grey part is not explained in the manuscript, which is mainly to describe the global properties for the project level.

The csml:entity and csml:process elements respectively represent an entity and a process in HFPNe. In csml:process, the entities involved in a process are represented via the csml:connector element, which denotes a connector in HFPNe. The csml:connector element connects the corresponding process and the involved entities by referencing the ids of csml:entity. The csml:fact element represents some of the properties of the modeling pathway that cannot be described in HFPNe. For example, the functionality of a pathway and the status of a dynamic simulation are useful in gaining an overall understanding of the pathway. This kind of information is represented as csml:fact.

Each of csml:entity, csml:process, csml:fact, and csml:connector has a type attribute indicating the semantic constraints on the meaning of each element. The type attribute has a value that is a class name hierarchically conceptualized in the Cell System Ontology (CSO) [19]. CSO is a formal ontology for biological pathway modeling with dynamics. The basic components of HFPNe – csml:entity, csml:process, and csml:connector – have their own biological properties, simulation properties, and view properties. The csml:biologicalProperty element is associated with the core terms defined in CSO to annotate biological properties such as cellular components and biological events. CSO also defines controlled vocabulary terms internal to the ontology. The csml:simulationProperty element is used to represent detailed simulation rules in HFPNe. The csml:viewProperty element comprises several elements for the visualization of pathway components, such as an image file, graphical shape, and geometrical position. This property is rarely included in other formats such as SBML[16] and CellML[17].

Additionally, in CSML, it is possible to create sub-models from the main model by using filtering and selection. For such sub-models, multiple views can be assigned. However, these features are not used in this work.

### TRANSPATH

The TRANSPATH database [20] is designed to provide information about the intracellular signaling pathways. The database presents such information on different abstraction levels of the reactions with all the required molecular information about post-modification processes and complexes. TRANSPATH offers molecular details of the signal flow from the cell surface into the nucleus, focusing on mammals such as humans, mice, and rats. The database contains information manually extracted from the literature, except for the 1,129 initial molecules imported from SWISS-PROT release 36. As of April 2007, TRANSPATH Professional release 8.1 contains about 74,000 molecules, over 25,500 genes and over 115,000 reactions collected from approximately 31,500 references [21]. In addition, each reaction is provided with a reliability value based on the quality of experimental data in the primary literature. In terms of both the quantity and quality of data collected, TRANSPATH is considered to be very valuable to researches in biochemistry and molecular biology.

### Summary

Modeling, visualizing, and simulating biological pathways are becoming increasingly important tasks for understanding dynamic cell systems. We have been developed an XML language, CSML, as an integrated framework for such tasks. This work provides a methodology for reconstructing information from the TRANSPATH database into the CSML format, one based upon the HFPNe modeling rules. A major challenge is to accurately translate the information from a pathway database to an exchange format that enables dynamic simulation with visualization. The CSML and its platforms [22-25] could be used to check whether the model is represented in a consistent way, it is amenable to quantitative simulation, and the pathway components are correctly located in cellular location. The individual reactions and combinations of reactions in TRANSPATH are evaluated by the HFPNe modeling rules and translated into CSML. In addition, the reconstruction could allow easy exchange and integration of signal transduction information, which will enhance the usability of the TRANSPATH database.

## Results

The full contents of the TRANSPATH database are supplied in the XML (Extensible Markup Language) format [26]. Hereinafter, we refer to the TRANSPATH database as TP; the term TP might be used to indicate the XML format itself whose tags are prefixed with "tp:." Based on the HFPNe modeling rules described in Methods section, we have implemented the converter Transpath2CSML that can reconstruct reactions and pathways defined in TP into CSML. During reconstruction, entities and processes deficient in TP but necessary for simulation-based models are inferred and added to create valid CSML models.

In the following sections, we describe the reconstruction results for the main components in TP: molecule, reaction, and pathway. The HFPNe modeling rules are developed at the reaction level in TP. For molecules including genes, we focused on the visualization.

Note that there are also elements common to the TP components, such as tp:creator, tp:updator, tp:name, tp:synonyms, and tp:comments. The tp:creator and tp:updator elements indicate who registered and updated the data, respectively. The tp:name and tp:synonyms elements denote the name of the component and its synonyms. The tp:comments element is a text-based comment for information that cannot be described in other tags. These tags are also mapped to the CSML format without any loss of information, although they are not shown in this paper.

### For molecules

The molecules in TP are hierarchically classified [20] such as basic, isogroup, family for proteins. This type is not distinguished from the name of the molecule without extracting the detail. Visualization can help interpret large quantities of complex data. For this, we propose the visualization method to fully reflect the relationship among molecule types.

**Figure 2:**
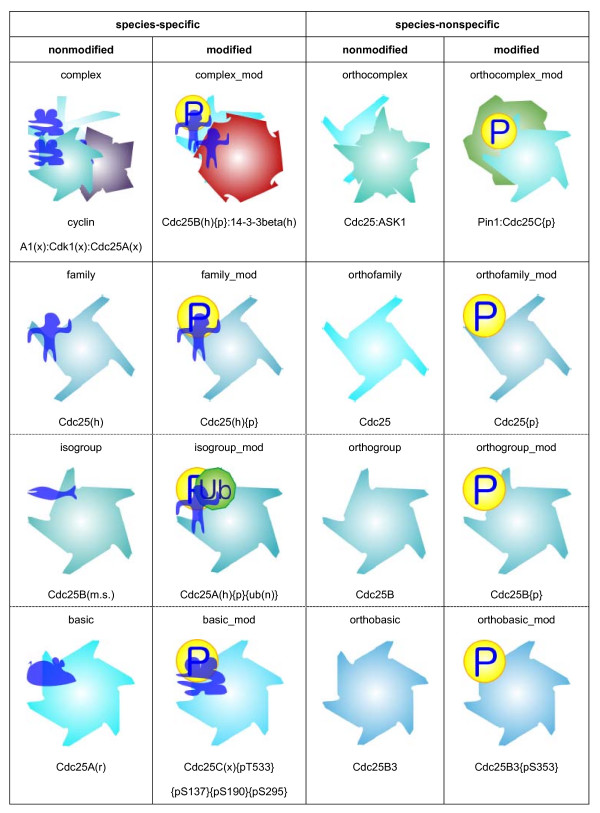
Graphics automatically generated for Cdc25 family proteins defined in TP.

Regular polygons are used for proteins. Each nonprotein is also depicted as an icon. Different numbers of line segments are used to distinguish families, isogroups, and basic types, i.e., 4, 5, and 6, respectively. Different line patterns of polygons are used to distinguish proteins at the level of an orthofamily. There are, in total, 952 orthofamily proteins in TP. Each orthofamily is assigned to a line pattern and color, and then the proteins belonging to the same orthofamily are assigned to similar colors during conversion. In addition, an mRNA and its product protein use the same color to indicate their relationship. We also designed icons representing different species and post-translational modification. These icons are attached to polygons on the fly during conversion. A complex protein is shown as composed of the graphics of its components. The generated graphics are saved in the SVG (Scalable Vector Graphics) format [27] and embedded into CSML. Currently both scalable and nonscalable image formats are supported in CSML. Figure 2 shows the automatically generated graphics of molecules categorized into the Cdc25 family.

**Figure 3:**
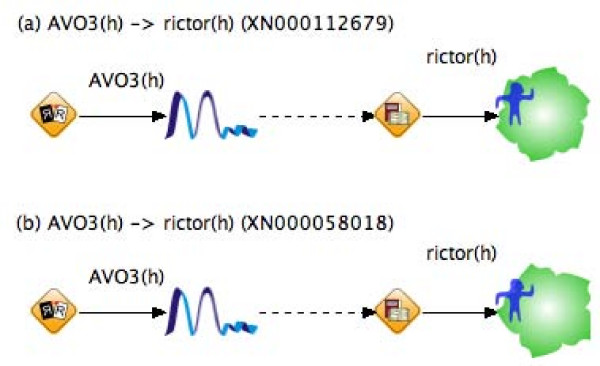
**The converted results showing the difference between molecule types**. (a) a basic type rictor(h) is shown as hexagon (b) an isogroup type rictor(h) is shown as pentagon.

From the graphic of a molecule, the type of molecule is easily identified. For example, in TP, two semantic type reactions, XN000112679 and XN000058018, have the same tp:name, AVO3(h) --> rictor(h), i.e., AVO3 expresses rictor in humans. The difference between the two reactions is the type of rictor, i.e., a basic type in XN000112679 and an isogroup type in XN000058018. Figure 3 shows the converted results for two reactions: (a) XN000112679 and (b) XN000058018. The output entity rictor is drawn as hexagon in (a) and pentagon in (b), which will reduce the effort required to extract the details of the molecule type.

### For reactions

We have defined the HFPNe modeling rules for the effect terms of the reactions in TP. Basically, a reaction in TP is converted into one or more csml:process elements in CSML. The participants in a reaction are converted into pairs of csml:entity and csml:connector elements. The type attribute of csml:connector indicates the role of an entity and whether it is involved as an input or an output in a process. The values are class names defined in CSO [19]: InputProcess as an input substrate, InputAssociation as an input activator, InputInhibition as an input inhibitor, and OutputProcess as an output product. The role of the participating entity is decided from the combination of the effect terms and the description of the entity in TP. The OutputProcess type connector may indicate the role of the process as an input.

The reactions in TP are mainly divided into three types: pathway step, molecular evidence, and semantic. The first two types are converted into simulatable processes in CSML, while semantic level reactions cannot be directly converted into processes since the details of the reactions are not given. However, a semantic level reaction may have the tp:pathway_step and tp:molecular_evidence elements, which store the related pathway step and evidence reactions, respectively. In such a case, it is possible to reconstruct a simulatable model. During conversion, default values are assigned to simulation related parameters defined in CSML. Otherwise, the semantic reaction is mapped to the csml:fact element in CSML, which is still useful for examining the result of the model simulation. For example, one semantic reaction describes that molecule A is increased after the stimulation of drug B via an unknown indirect regulation. Because there are no details of the reaction, the participants of the reaction (A and B) and any effect terms will be saved into csml:fact. In a simulation tool such as Cell Illustrator [24,25], the user can check the correspondence between the simulation results of the whole pathway and the experimental results described in csml:fact for the reaction.

**Figure 4:**
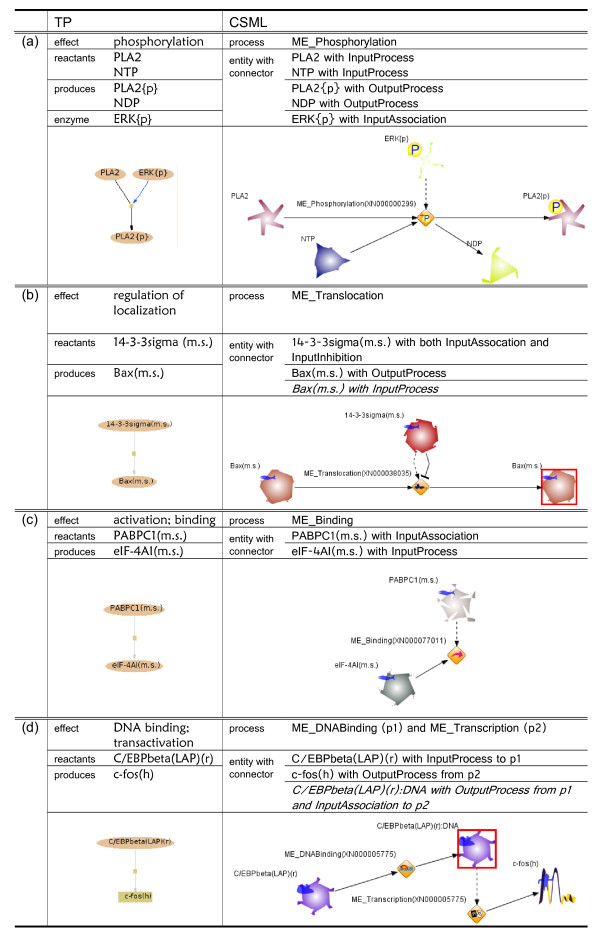
**Conversion results of reactions in TP**. (a) Rule 1: TP reaction (XN000000299) PLA2 + NTP --ERK {p}--> PLA2{p} + NDP (b) Rule 2: TP reaction (XN000038035) 14-3-3sigma(m.s.) --> Bax(m.s.) (c) Rule 3: TP reaction (XN000077011) PABPC1(m.s.) --> eIF-4AI(m.s.) (d) Rule 4: TP reaction (XN000005775) C/EBPbeta(LAP)(r) --> c-fos(h).

**Figure 5:**
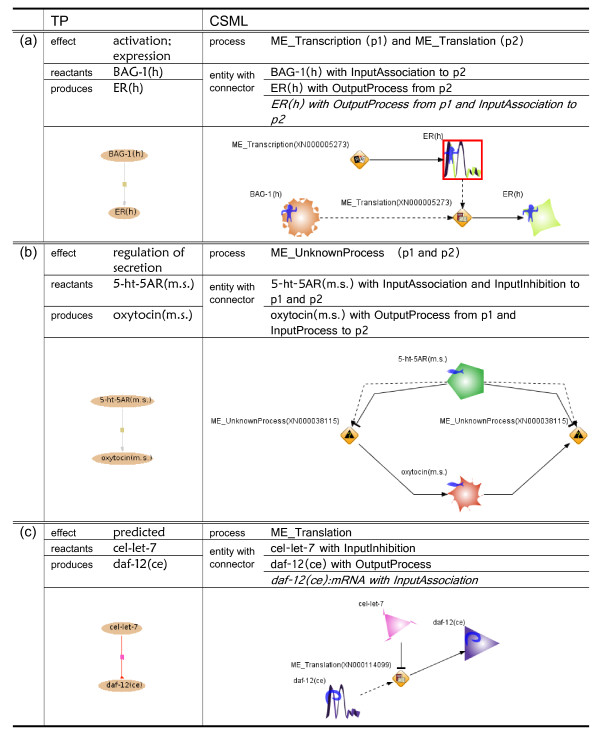
**Conversion results of reactions in TP (continued)**. (a) Rule 5: TP reaction (XN000005273) BAG-1(h) --> ER(h) (b) Rule 6: TP reaction (XN000038115) 5-ht-5AR(m.s.) --> oxytocin(m.s.) (c) Rule 7: TP reaction (XN000114099) cel-let-7 --/ daf-12(ce).

The conversion results based on the HFPNe modeling rules described in Methods section are shown in Figure 4 and Figure 5. Because of the limitation of space, the results are divided into two figures. We have selected one reaction for each group of rules as an example and provide the conversion result of the reaction. The left and right columns list the element names and their corresponding values in TP and CSML, respectively. The last row shows a drawing by PathwayBuilder [21] for TRANSPATH and that by Cell Illustrator [24,25] for CSML. PathwayBuilder uses the following legend for modeling: an oval with a name for proteins; rectangle with name, genes; small rectangle with no name, reactions; black line, mass flow; grey line, activation; red line, inhibition; and blue line, catalysis. In the Cell Illustrator drawing, the icons of the processes are also defined in CSO with their corresponding names as predefined instances. After conversion, the inferred entity that is necessary for simulation but missing in the original TP model is represented in *italics *and emphasized with a red box in the Cell Illustrator drawing. However, some effect terms that are assigned to pathway steps or molecular evidences in the original TP are ambiguous and cannot be directly used in the CSML simulation model. For example, as in Figure 4(b) and Figure 5(b), the effect term that includes "regulation" cannot clarify the role of a participant, e.g., whether it activates or inactivates the event. Therefore, we must add two connectors for the participant.

### For pathways

**Figure 6:**
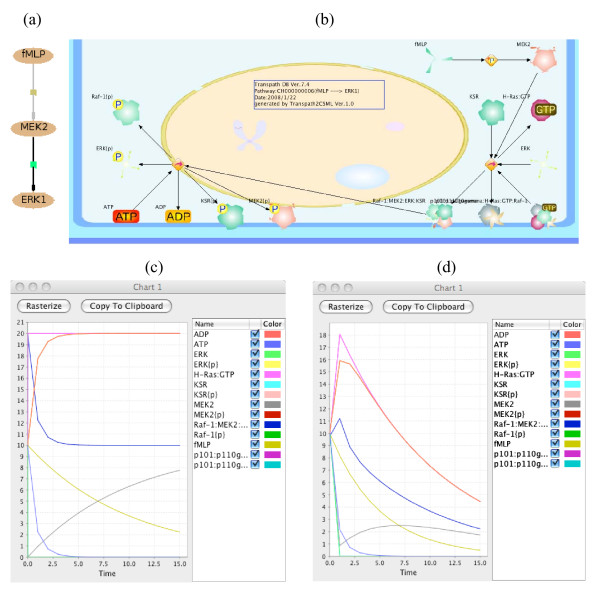
**Conversion results and simulation based on CH000000006, a pathway in TP**. (a) PathwayBuilder view from TRANSPATH. (b) Cell Illustrator view after conversion: The pathway is loaded into CI and drawn by default layout algorithm. (c) Simulation results with assigned parameters during conversion in Cell Illustrator. The chart shows the changes of molecule concentration along with those of time. The y-axis shows relative molecules' concentrations without units. The x-axis shows the changes of time. The needed values are automatically assigned during conversion for basic usage. (d) Simulation results with adjusted parameters in Cell Illustrator. The same notations are used as (c). The user can adjust the values during investigation and the results such as the changes of concentration with time will be saved into CSML. Note that this chart does not correspond to the experimental results.

A pathway is considered as a logical combination of reactions. The tp:pathway element, which consists of reactions and molecules, is converted into one csml:project element including one csml:model element. During conversion, we first consider pathway level reactions. If there are no pathway level reactions, evidence level reactions are considered. In addition, reactions related to a specific molecule as upstream and downstream are also used to build a pathway in CSML. As an example, we selected a pathway that fMLP activates ERK1 via the predominant MEK isoform in human neutrophils, MEK2 in TP. The pathway consists of two reactions XN000015421; fMLP ---> MEK2 (activation; phosphorylation) and XN000000703; MEK2 ---> ERK1 (activation; phosphorylation). Figure 6 shows (a) the PathwayBuilder view from TRASPATH, (b) Cell Illustrator view after conversion, (c) and (d) simulation results with default and adjusted parameters, respectively.

## Discussion

We are developing a systematic translation method for generating dynamic pathway models with visualization in CSML. We have suggested a formal mechanism that combines Petri nets modeling and the semantics in each biological reaction. The modeling rules generate a simulatable model for the whole pathway as a combination of processes as well as for an individual process. Our modeling rules can be applied to semi-structured information stored in TP.

When translating data, we have first observed that TP has an inconsistent and incomplete representation. In TP, the roles of the molecules in tp:reactants and tp:produces are changed along with the effect terms described in the tp:effect element. This inconsistency occurs because the effect terms reflect the descriptions in the literature. The relationship between tp:reactants and tp:produces is similar to that between a subject and an object in English grammar. Therefore, it does not reflect the biological relationship in a reaction. For example, from the sentence "A activates the binding of B," A is defined as a reactant and not an enzyme. This kind of description makes consistent interpretation impossible.

Secondly, TP is a database for static pathway models and generates a static drawing with a high level of abstraction, which is useful in providing a broad overview of pathways; moreover, this representation style is familiar to biologists. However, it increases the effort required to extract the essential information to generate a simulatable model. The implicit representation is shown as follows:

• The modification information for the molecule is mixed together in the name of the molecule in tp:molecule.

• The locations where the reaction has been shown indicate places including the cell line, stage, organ, and species used in the original literature, which are described all in tp:locations in tp:reaction. CSML separately describes the tissue type, cell type, and organism (species) as biological properties.

• Cellular compartment for the molecule and reaction is important to locate them at biologically meaningful locations in a cell. For example, the effect term "translocation" indicates a regulated transfer of a signaling molecule from one location to another location. It will improve the understanding of the pathway if the cellular compartments are described. However, TP does not explicitly define this information.

To improve the exchange and integration capabilities with the successive releases of TRANSPATH, we suggest that TRANSPATH uses a common vocabulary to annotate data such as tp:effect. Mature core terms defined in CSO for biological properties such as biological events and cellular locations will be useful because CSO includes most of the external references referred in BioPAX [18] for controlled vocabularies and reorganizes them for system dynamics.

In this work, we implemented rules for the automatic generation of icons for molecules, reflecting the species, modification, and subsumption relationship in the hierarchy of the molecules. The visualization could be improved by using a description in TP as follows:

• The similarity between proteins, and between an miRNA and its precusor pre-miRNA can be indicated by using similar line patterns. It could be supported by tp:klass, the classification stretches over all molecules, and tp:go, the GO terms from molecular functions and biological processes.

• The stroke widths of connectors reflect the quality of the experimental results in the primary literature, ranging from 1 to 5 in tp:quality (highest to lowest reliability with regard to biological relevance).

## Conclusion

We used a systematic method to reconstruct dynamic signaling pathways from static pathway databases. We identified a set of rules for HFPNe modeling in terms of biological processes. The basic methodology for these rules can be applied to other similar databases to build dynamic pathways. The conversion results are saved in CSML and can be used for further biological investigation, e.g., conversion to other formats for use with different visualizing tools and different simulating tools. The proposed methods were applied to the TRANSPATH professional 8.1. During our research, TRANSPATH 8.4 is released with more data, but with no changes of the format. The converted results in CSML are available for both professional (TRANSPATH 8.4) and academic (TRANSPATH 7.4) users via the Cell Illustrator Online (CIO) [28]. TRANSPATH 7.4 in CSML is free to all academic users upon registration. It is possible to search which entities (including genes and proteins modified/complex) are related to a queried entity and which processes include the queried entity. The part of or all results can be used to build a pathway. Each pathway stored in TRANSPATH is provided as one project in CIO.

## Methods

All the signaling pathways in TP are modeled as bipartite graphs with molecules and reactions as two disjoint node sets. Each molecule is linked to the reaction in which it participates. Both molecules and reactions are hierarchically organized [20].

TP mainly consists of four components: pathway, reaction, molecule, and gene. The pathway component stores a group of chains and pathways, which consist of a set of reactions. The reaction component stores the cellular processes of the molecule and gene components, e.g., p53 binds to Mdm2. The molecule component describes the protein and small molecule information, e.g., p53 protein and Mdm2 protein. The gene component stores the gene information, e.g., *p53 *gene and *MDM2 *gene.

### Visualization method for molecules in TRANSPATH

In TP, molecules that are gene products are separated from genes. Molecules are basically divided into proteins and nonproteins as shown in Table 2. The proteins are further divided into 16 types depending on whether the molecule is species-specific or not, modified or not, and complex or not. The noncomplex proteins in rows 4–6 in Table 2 are categorized into three types: basic, isogroup, and family. "Basic" represents an individual polypeptide having a sequence and a molecular weight, e.g., the proteins Cdc25B1 and Cdc25B2 in humans. All the products from one gene are grouped into an "isogroup," e.g., the Cdc25A and Cdc25B protein groups in humans. The "family" is a more general "isogroup", e.g., the Cdc25 proteins in humans. There exists a molecular hierarchy among families, isogroups, and basics in decreasing order of granularity. The prefix "ortho" is attached for species-nonspecific molecules. The suffix "_mod" is added to represent a chemical modification such as phosphorylation. The complex type has "complex" in its name.

**Table 2:** Types of molecules defined in TP.

(a) Proteins			
**species-specific**		**species-nonspecific**	
	
**nonmodified**	**modified**	**nonmodified**	**modified**

Complex	complex_mod	orthocomplex	orthocomplex_mod

Family	family_mod	orthofamily	orthofamily_mod
Isogroup	isogroup_mod	orthogroup	orthogroup_mod
Basic	basic_mod	orthobasic	orthobasic_mod

(b) Nonproteins			
group (XOR), mRNA basic, miRNA basic, pre-miRNA basic, small molecule, and other			

The molecule type is stored in the tp:type element. The relationships between molecules are described in tp:members for any subfamily and in tp:groups for any superfamily of a molecule. For example, PDGF (orthofamily) has PDGF A (orthogroup) as a member and belongs to the PDGF/VEGF family (orthofamily) superfamily. In the case of a species-specific protein, Ret(h) (isogroup) has Ret-1(h) and Ret-2(h) (basic) as its members and belongs to the Ret (orthofamily) and Src(h) (family) superfamilies. If the molecule is a complex, the components of the complex are described in tp:stateofs.

Unfortunately, the post-translational modification (PTM) of a molecule is not explicitly defined in TP, but is, instead, described by a human-readable identifier in tp:name. Detailed information regarding PTMs is represented with braces, e.g., {p} for phosphorylation at an unknown position and {pT387} for phosphorylated threonine at position 387. The name of a complex uses a colon, e.g., A:B. In addition, the species information is also included in tp:name by using parentheses, e.g., (h) for *Homo sapiens *and (m) for *Mus musculus*, although there is the tp:species element for describing species.

The molecular hierarchy would be useful for pathway visualization, providing a qualitative understanding of the information content. In this work, we focused on developing a method to graphically represent such information in a manner that users can understand without extracting the details of the tp:molecule structure as much as possible. We choose three features to be represented: the subsumption relationship among noncomplex proteins, species, and modification. For visualization of molecules based on the types shown in Table 2, molecules are depicted as icons with different visual properties: the number of line segments, line patterns, and colors.

### Modeling rules for reactions in TRANSPATH

In TRANSPATH, a reaction describes any kind of interaction between signaling entities (molecules or genes) in signaling or regulatory events. A reaction is the basic building block of the pathway.

There are four kinds of reactions in TP: molecular evidence, pathway step, decomposition, and semantic [29]. The "molecular evidence" reactions are extracted from scientific publications by manual curation reflecting the results of experiments. Because the same reaction may be dealt with using different experimental setups, these reactions contain some redundancy and hetero-species data. Such reactions are condensed into nonredundant pathways and summarized into one reaction step; this is called the "pathway step" reaction as a more general level. The "semantic level" reaction is the most abstract reaction, providing a broader shortcut overview of the signal transduction pathways without biochemical details. For example, a DNA binding reaction can be represented as "COUP-TF2(h) --> CRBPII(m)" (id XN000007077) on the "molecular evidence" level and "COUP-TF2 (NR2F2) --> CRBPII" (id XN000059803) on the "semantic" level. As a result, in the hierarchy of reactions, a "semantic level" reaction may consist of multiple "pathway step" reactions, and each "pathway step" reaction, in turn, consists of multiple "molecular evidence" reactions. The "decomposition" reaction explains the mechanism of a reaction and identifies the reacting molecules of reactions that occur in complexes.

The main features of a reaction are the explanation of a detailed biochemical reaction and the molecules involved in the reaction such as tp:effect and tp:reactants.

The tp:effect element provides the details of reaction mechanisms such as phosphorylation and cleavage; TP defines 149 single effects. A reaction may be annotated by more than one effect term. However, combinations of effect terms, e.g., "binding; phosphorylation," have no explicit definitions. Furthermore, the difference between "binding; phosphorylation" and "phosphorylation; binding" is unclear. Thus, we disregard the order of terms described in tp:effect. As a result, the above two terms are considered equivalent. There are, in total, 521 different effect terms used to explain reactions in TP. In this work, we consider the 50 most frequently used effects for conversion, which occupy over 97% of all reactions, as shown in Table 3; the other effect terms are rarely used in TP. The remnant effect terms are mapped to unknown processes in CSML, though they will be converted with a similar methodology described in this section.

**Table 3:** The top 50 effect terms of reactions with "pathway step" and "molecular interaction" type in the TRANSPATH database.

**No.**	**Effect**	**Rule**	**#(%)**	**No.**	**Effect**	**Rule**	**#(%)**
1	binding	1-1	28411 (29.5%)	26	sumoylation	1-1	89486 (92.8%)
2	expression	5-1	53378 (55.4%)	27	decrease of phosphorylation	3-1	89846 (93.2%)
3	DNA binding	4-1	59299 (61.5%)	28	dephosphorylation	1-1	90183 (93.5%)
4	phosphorylation	1-1	64800 (67.2%)	29	increase of binding	2-1	90516 (93.9%)
5	activation	2-1	68829 (71.4%)	30	predicted	7	90844 (94.2%)
6	increase of abundance	2-1	72516 (75.2%)	31	binding; phosphorylation	1–2	91130 (94.5%)
7	DNA binding; transactivation	4-1	75753 (78.6%)	32	translocation	1-1	91397 (94.8%)
8	decrease of abundance	3-1	77255 (80.1%)	33	acetylation	1-1	91629 (95%)
9	increase of phosphorylation	2-1	78570 (81.5%)	34	binding; oligomerization	1–2	91853 (95.3%)
10	regulation of abundance	6-2	79728 (82.7%)	35	transrepression	2–3	92052 (95.5%)
11	inhibition	3–4	80790 (83.8%)	36	regulation of phosphorylation	2–4	92247 (95.7%)
12	regulation of activity	6-1	81810 (84.9%)	37	decrease of secretion	3-1	92425 (95.9%)
13	regulation of localization	2–4	82806 (85.9%)	38	activation; binding	3-2	92585 (96%)
14	transactivation	2-1	83507 (86.6%)	39	automodification; phosphorylation	1-1	92734 (96.2%)
15	DNA binding; transregulation	4-3	84120 (87.3%)	40	degradation	1-1	92866 (96.3%)
16	increase of secretion	2-1	84721 (87.9%)	41	exchange	1-1	92995 (96.5%)
17	regulation of protein activity	6-1	85280 (88.5%)	42	oligomerization	1-1	93114 (96.6%)
18	DNA binding; transrepression	4-2	85814 (89%)	43	decrease of protein stability	3-3	93226 (96.7%)
19	cleavage	1-1	86327 (89.5%)	44	hydrolysis	1-1	93327 (96.8%)
20	activation; increase of phosphorylation	2-2	86836 (90.1%)	45	dissociation; phosphorylation	1–3	93420 (96.9%)
21	activation; expression	5-2	87309 (90.6%)	46	degradation; destabilization	3-3	93510 (97%)
22	ubiquitination	1-1	87766 (91%)	47	regulation of secretion	6-2	93593 (97.1%)
23	binding; regulation of localization	1–2	88216 (91.5%)	48	activation; increase of abundance	2-2	93673 (97.2%)
24	processing	1-1	88665 (92%)	49	increase of processing	2-1	93753 (97.3%)
25	binding; regulation of protein activity	1–2	89094 (92.4%)	50	decrease of binding	3-1	93832 (97.3%)

**Figure 7:**
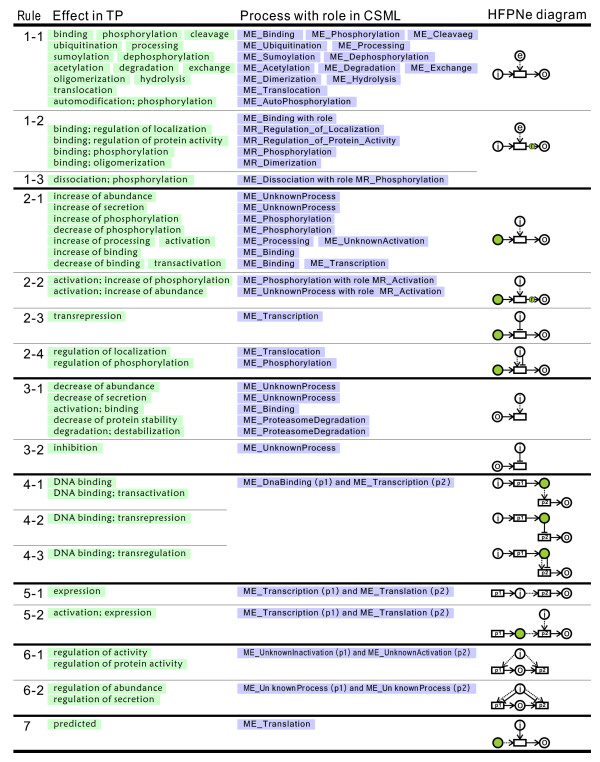
**The HFPNe modeling rules to convert from TP reactions to CSML processes**. The columns show the rule number, the effect terms used in TP, the corresponding molecular event in CSO, and the HFPNe diagram. In the third column, the molecular event is prefixed with "ME_" and the role of the process is with "MR_." In the HFPNe diagram, a circle indicates one or more participants involved in a process: i for tp:reactants, o for tp:produces, and e for tp:enzyme in TP. A rectangle describes a process. The inferred entities for the corresponding process are shown in colored circles. If a process has a specific role, the output connector is shown with a colored circle.

These 50 terms are used to annotate the effects of reactions (pathway step and molecular evidence type), and occupy 97.3% of the total number of reactions (96404). The No. column lists the frequency order of the effect terms; the Effect terms; the Rule number in Figure 7; and the #(%) column, the accumulated frequency and its percentage in parentheses.

In CSML, a process is a biological event that affects the kinetics of a simulation. In addition, a process defines an elementary reaction, and not a combination of reactions. CSML refers to 274 biological events defined in CSO to annotate the type attribute of csml:process. Biological events in CSO are classified into cellular, organism, molecular, and physiological events. In this work, the CSO terms defined for molecular events are used to describe the reactions in TP.

There are several difficulties encountered when developing modeling rules for mapping between tp:effect and the type attribute of csml:process. First, tp:effect uses terms to place more importance on the results of a reaction than on the reaction itself. Generally, the results of a reaction and the reaction itself are not easily distinguishable. For example, a "decrease of secretion" could arise from the existence of another protein or as a consequence of arbitrary reactions such as the inhibition of intracellular protein hydrolysis. Thus, terms such as "increase of secretion" and "decrease of amount" are intended to represent the dynamic behaviors in TP. In CSML, such effects are handled by connectors (OutputProcess in CSO) that link a process to an output entity, which hold the detailed role of the process. Secondly, one effect term in TP may imply several steps of molecular events in CSO. For example, "expression" is defined as a transfer of the information encoded in DNA into a protein; reactions with effect expression include several steps: transcription, splicing, capping, and translation in TP. In CSO, expression is represented as two processes: DNA-mRNA transcription and mRNA-protein translation. Thirdly, on the other hand, serialized effect terms in TP may mean one step of a molecular event in CSO. For example, the effect term "phosphorylation; automodification" in TP is mapped to autophosphorylation in CSO. Thus, a direct mapping of an effect term in TP to a molecular event in CSO is not available.

Molecules involved in a reaction are described in tp:reactants, tp:produces, tp:enzyme, and tp:inhibitor, whose names indicate the roles of the molecules. However, the description in TP is not consistent in the sense that the role of each participant is altered depending on the reaction. For example, the reaction (XN000004663) "NO --/ Caspase-1(h)" means that NO semantically inhibits an unknown activity of Caspase-1. In this reaction, Caspase-1 is a reactant and NO is an inhibitor, rather than a product and a reactant defined in TP, respectively.

For conversion, we must carefully define the roles of participants depending on the effect terms. In addition, whether the role of an enzyme in a reaction is that of an activator or an inhibitor is not explicitly described in TP. If it is difficult to identify the roles of enzymes, we add two connectors: an association connector (as an activator) and an inhibitor connector (as an inhibitor) between the enzyme and the reaction. By default, the inhibition connector will work when the threshold of the connector is less than the concentration of the enzyme. As to which one correctly corresponds to experimental results – that will be examined via simulation. The speed of one connector is set to zero while another one works.

For a kinetic reaction, the stoichiometric coefficients are also not explicitly represented in TP, but tp:name of a reaction contains these values. For example, the reaction (XN000000000) is described as "2ErbB1 + EGF --> EGF(ErbB1)2" in tp:name. In this case, we extract the coefficients from tp:name.

There are also elements to indicate the directionality and reversibility of a reaction. The tp:direct element notifies whether the reaction is direct or not. If interactions may contain yet unidentified steps between the reactants and products at the semantic level, these are indirect interactions. The tp:reversible element is used to denote whether a reaction is reversible or not. During simulation, the speed in both directions may be different. Therefore, a reversible reaction is mapped to two different processes in CSML.

For implementation, we proposed modeling rules for representing the semantics of reactions in terms of HFPNe modeling. Inference requires an overall interpretation from the definition of the effect term and the molecule types described in tp:reactants and tp:produces.

Figure 7 shows the modeling rules for mapping from 50 TP effect terms to CSO molecular events. The columns show the rule number, the effect terms used in TP, the corresponding molecular event in CSO, and the HFPNe diagram. In the third column, the molecular event is prefixed with "ME_" and the role of the process with "MR_." In the HFPNe diagram, a circle indicates a participant involved in a process: i for tp:reactants, o for tp:produces, and e for tp:enzyme in TP. As shown in the diagram, the roles of the participants are not consistent but are changed depending on the effect term. A rectangle represents a process. The inferred entities are shown in colored circles. If a process has a specific role, the output connector is represented with a colored circle, e.g., R1-2 and R1-3. The 16 modeling rules are grouped together based on the HFPNe diagram. For example, R1 means a group consisting of rules R1-1, R1-2, and R1-3. We use this notation hereinafter.

The R1 group shows a reaction catalyzed by an enzyme. The molecules in tp:reactants and tp:produces are mapped to input and output entities in CSML. If tp:enzyme is defined, the enzyme is connected to a process via association connectors. R1-2 and R1-3 are basically mapped to the same HFPNe model, but differ in terms of the molecular event and its role. All effect terms in R1-2 are mapped to the same event "ME_binding," but each process has a different role with the "MR_" prefix. For example, "binding; regulation of localization" is mapped to the event "ME_Binding," whose role is "MR_Regulation_of_Localization."

The R2 and R3 groups are basically the same HFPNe model as R1. However, depending on the effect terms in TP, the participants play different roles. Furthermore, in order to generate dynamic pathway models, some participants may have to be inferred during conversion.

In R2, the molecules in tp:reactants and tp:produces are mapped to enzymes and output entities in CSML, respectively. For example, the effect term in the reaction (XN000027773) "SHP-2(h) --> STAT5A(h){pY694}" is "increase of phosphorylation." This type of reaction in TP usually omits the input of the reactions. In the above example, STAT5A(h){pY694} is a phosphorylated molecule, and there is no pre-phosphorylated one before the process. If there is an explicit need for input entities, they are inferred. As described before, there is no explicit notation for the role of an enzyme, i.e., as an activator or an inhibitor. Basically, the role of an enzyme is assumed to be that of an activator. If the effect term contains "repression" or "inhibition," then the role of enzyme is that of an inhibitor. In R2-3, transrepression is an inhibited reaction meaning the repression of a gene by a transcription factor in TP. If the effect term "regulation" is used, we assumed that the enzyme plays two roles – both that of an activator and an inhibitor. In R2-4, both association and inhibition connectors are used to link the enzyme and process.

R3 is different from R2 in the sense that the molecules in tp:produces are mapped to input entities and not to output entities. In R3-1, the effect term in the reaction (XN000038092) "5-HT-2B(m.s.) --> Bax(m.s.)" is "decrease of abundance," i.e., the amount of Bax is decreased by 5-HT-2B. The effect term "activation; binding" is interpreted as that the binding activity of tp:produces is enhanced by tp:reactants. R3-2 is for the effect term "inhibition." Because the kind of process that is inhibited is not explicitly stated in TP, this reaction is mapped to an unknown process.

The effect terms in the R4 group are all related to DNA binding whose definition is "binding of a protein to the promoter/enhancer of a gene" in TP. In the description of the reaction (XN000005775) "C/EBPbeta(LAP)(r) --> c-fos(h)," tp:reactants is a protein and tp:produces is a gene. This means that the product of the DNA binding of a protein activates the transcription of a gene. From the definition of DNA binding in TP, this reaction implies two processes: DNA binding and then transcription. In HFPNe modeling, the complex of DNA and a protein (in tp:reactants) is introduced as a regulator of transcription. The regulation is identified with the last effect term such as transactivation and transrepression.

The R5 group is for gene expression and is defined as "transfer of information encoded in the DNA into the protein" in TP. For HFPNe modeling, two molecular events, transcription (p1) and translation (p2), are used to represent expression. The difference between R5-1 and R5-2 is in the role of tp:reactants because of the effect term "activation" in R5-2. In R5-1, tp:reactants is mapped to a product generated by a transcriptional process (p1). On the other hand, in R5-2, tp:reactants is an activator that enhances the transcriptional process. In this case, the product of transcription (p1) must be inferred.

The R6 group is for effect terms combining abundance/activity and regulation. These terms can be used to represent a circumstance in which the target protein abundances are increased/decreased by antibodies, e.g., antibodies interacting directly with protein active sites or blocking the access of substrates to the active sites. For these terms, we use two HFPNe models. In R6-1, the enzyme regulates the activity of the product as the activator of two processes – unknown inactivation (p1) and unknown activation (p2) – in which the product takes part. On the other hand, in R6-2, the enzyme regulates two unknown processes via two connectors. R6 is different from R2 in the sense that the terms in R2-1 describe an activator of a process, including the unknown process, and the terms in R2-4 describe a regulator for a specific process such as localization. The detailed mechanism is usually described in tp:comment. The contents of tp:comment may be useful for building a better model.

Rule 7 is a specific type for the effect term "predicted," whose definition is "*in silico *predicted interactions of an miRNA with the complementary site of a target mRNA." This term indicates that miRNAs repress the translation of target mRNAs. For HFPNe modeling, the reactant (miRNA) inhibits the translational process of the product (mRNA) and the input entity (pre-translational mRNA) must be inferred.

### Conversion of Pathways

The tp:pathway element describes a pathway and may contain any number of chains as pathway components. The chains are sets of consequent reactions that are pathway steps. The chains are classified into three types: chain, evidence chain, and metabolic chain. When a sequence of reactions has been reported by one primary paper, the chain is labeled as an "evidence chain." The "metabolic chain" means that the chains are constructed from metabolic pathway step reactions. Each type of chain is also defined as a pathway in TP. We call pathways that consist of chains as normal pathways to differentiate them from the three chain-type pathways.

In TP, there are 1221 pathways in total. The number of each pathway type is 119, 589, 356, and 159, in the order of normal pathway, chain, evidence chain, and metabolic chain. The tp:pathway element may contain tp:chains when it is a normal pathway to describe the chains. In the tp:pathway element, the tp:reactions_involved and tp:molecules_involved elements specify the reactions and molecules involved in the pathways, respectively.

Each pathway in TP is mapped to one CSML file which contains one csml:project that has one csml:model. Because tp:chains has a certain meaning for a group of reactions, tp:pathway and tp:chains are mapped to csml:fact, which means a nonsimulatable biological fact. The involved reactions and molecules are mapped to the respective components of csml:processSet and csml:entitySet. Each reaction involved in the tp:pathway will be mapped to one HFPNe modeling rule depending on its effect terms, as described in the previous section.

## Authors' contributions

MN conceived the basic idea, developed the conversion rules for the TP, implemented the converter of TP, and wrote the draft of the manuscript. AS developed and implemented the routine of automatic graphics generator of mRNAs and proteins in TP. EJ, AS and MN evaluated the result of the converted result and checked the behaviors of the converter. The part of introduction is prepared by CL. The whole contents are revised by EJ and most of the snapshot of figures are taken by AS. SM supervised the whole study. All authors read and approved of the final manuscript.
